# Comorbidity prevalence of degenerative lumbar scoliosis in lumbar spinal stenosis: age-related radiological and symptomatological characteristics in a large sample

**DOI:** 10.3389/fneur.2026.1723983

**Published:** 2026-04-09

**Authors:** Yudong Zhao, Junjie Ma, Yi Zhao, Zhiqian Luo, Hongbin He, Wang Luo, Zhenliang Zhang, Zhuoran Sun, Siyu Zhou, Weishi Li

**Affiliations:** 1 Orthopaedic Department, Peking University Third Hospital, Beijing, China; 2 Peking University Health Science Center, Beijing, China; 3 Beijing Key Laboratory of Spinal Disease Research, Beijing, China; 4 Engineering Research Center of Bone and Joint Precision Medicine, Ministry of Education, Beijing, China; 5 Nanchong, Sichuan, China; 6 The First Affiliated Hospital of Hebei North University, Zhangjiakou, Hebei, China

**Keywords:** Berjano classification, coexisting, coronal imbalance, degenerative lumbar scoliosis, lumbar spinal stenosis, prevalence, sagittal imbalance, spinal deformity

## Abstract

**Background:**

The aging population with lumbar spinal stenosis (LSS) is leading to a higher prevalence of coexisting conditions, particularly degenerative lumbar scoliosis (DLS). However, large-scale studies on the prevalence of DLS in LSS patients are limited. The surgical management of LSS with DLS is controversial. Berjano’s DSD classification provides a framework based on the relationship between the responsible segment and scoliosis apex. Understanding the distribution of these patterns is an essential step toward elucidating the clinical characteristics of LSS patients with coexisting DLS.

**Methods:**

DLS was diagnosed according to the definition of a coronal curvature (main lumbar curve) of ≥10°. Patients were classified according to Berjano’s DSD classification. Spearmann correlation tests were conducted to examine the relationships between radiographic parameters and symptom scores in patients with coexisting conditions.

**Results:**

A total of 443 patients (157 males and 286 females, mean age 65.5 ± 7.5 years, mean Cobb angle 18.0 ± 7.9°) met the criteria for DLS (Cobb angle ≥ 10°), leading to a coexisting prevalence of 26.2%, ranging from 12.0 to 35.6% increasing with age. Patients with Berjano type I, type II, type III, type IVa, and type IVb account for 35.0, 38.8, 13.3, 12.0, and 9.0%, respectively. In patients with LSS and comorbid DLS, VAS LBP and ODI scores were both correlated with sagittal parameters. VAS LBP was significantly correlated with the Cobb angle (*p* = 0.001), while ODI scores showed significant correlations with the C7 plumb line to central sacral vertical line (C7PL-CSVL) (*p* = 0.038). Meanwhile, significant correlations were observed between sagittal parameters, and the Cobb angle and C7PL-CSVL, respectively. The incidence of sagittal imbalance (SVA > 50 mm) in LSS patients with DLS was 41.1%, which increased with the severity of coronal imbalance.

**Conclusion:**

The prevalence of DLS in LSS patients is 26.2%, increasing with age. Berjano type II represents the highest proportion (38.8%). Spinal deformity significantly correlates with clinical symptoms, with coronal and sagittal deformities being interrelated.

## Introduction

Lumbar spinal stenosis (LSS) is a prevalent degenerative spinal disorder causing chronic low back pain, leg pain and neurological dysfunction in the elderly population (1, 2). Its core pathology lies in the chronic neural injury caused by long-term, progressive mechanical compression of neural structures within the spinal canal, including the cauda equina and nerve roots. When conservative treatment fails, surgical decompression is the standard treatment to relieve neural compression and improve symptoms (3). However, in aging LSS patients, multiple degenerative changes usually coexist, such as lumbar disc herniation (4), spondylolisthesis (5), and instability, presenting significant challenges for neurosurgical diagnosis and treatment (6–9).

Among the coexisting pathological changes, degenerative lumbar scoliosis (DLS) is one of the most common and significant conditions, introducing a three-dimensional structural deformity including coronal scoliosis, sagittal imbalance, and vertebral rotation (8, 9). For LSS patients, these structural changes can further compromise the already stenotic spinal canal, thereby potentially exacerbating the neural injury and clinical symptoms. Since patients with LSS and coexisting DLS are subjected to the dual impact of both neural compression and spinal deformity, conventional treatment philosophies based on patients with isolated LSS may not be applicable, which renders their management and surgical treatment a controversial topic (10, 11). However, before these controversies can be further addressed, a thorough understanding of the clinical characteristics of this specific patient population is essential. However, in existing studies, significant heterogeneity was observed, with the prevalence of concomitant DLS reported to be 11.6–21.9% in LSS patients (10–14). To further evaluate the effect of such structural pathology on the neurological functions, a larger sample size-based study exploring is necessary.

Therefore, the purpose of this study was to leverage a large, single-center database to comprehensively investigate the clinical and radiographic characteristics of surgical LSS patients with coexisting DLS. Specifically, we aimed to: (1) determine the precise age-related prevalence of DLS in this population; (2) describe the distribution of deformity patterns using the Berjano classification system; and (3) elucidate how the severity of spinopelvic deformity contributes to the patient’s perceived burden of neural injury, as measured by functional disability and neurological symptoms.

## Methods

### Study design

This study was a single-center retrospective study. The study protocol was approved by the Medical Ethics Committee of Peking University Third Hospital (No. LM2024304), ensuring adherence to the ethical standards of the Declaration of Helsinki.

### Study population

A total of 1,770 patients with LSS who were scheduled for surgical intervention at our institution between January 2018 and September 2020 were retrospectively enrolled. LSS Patients scheduled for surgical intervention at our institution between January 2018 and September 2020 were retrospectively enrolled. The diagnosis of LSS was based on clinical symptoms, including low back pain (LBP), leg pain, numbness, and intermittent claudication, and was confirmed by magnetic resonance imaging (MRI) performed on a 3.0 T scanner with slice thicknesses of 4.0 mm. The inclusion criterion was patients older than 45 years diagnosed with LSS. The exclusion criteria were: (1) absence of complete preoperative clinical or radiographic data (*n* = 0); (2) prior spinal surgery treatment (*n* = 3); (3) concomitant thoracic spinal stenosis or neuromuscular disease (*n* = 45); (4) history of severe hip or knee joint disease (*n* = 0) and (5) history of previous scoliosis, tumor, or infection (*n* = 33). After applying these criteria, a total of 1,689 patients were included in the final analysis.

### Clinical evaluation

The clinical data for all participants were obtained from our hospital’s inpatient information system. The demographic data including age and sex were collected. Clinical symptom assessments were conducted using the Oswestry Disability Index (ODI) score, visual analogue scale (VAS) for low back pain (LBP), and VAS for leg pain.

### Radiographic measurements

Radiographic assessments were performed based on preoperative full-length standing whole-spine X-rays obtained under a standardized weight-bearing protocol (with patients in a free-standing position, knees locked, and arms flexed at approximately 30° to avoid superimposition).

Coronal radiographic parameters included: (1) Cobb angle: measured as the angle between the superior endplate of the upper-end vertebra and the inferior endplate of the lower-end vertebra of the main lumbar curve; and (2) C7 plumb line to central sacral vertical line (C7PL-CSVL): the horizontal distance from the C7 plumb line to the center of the sacral superior endplate, with values to the left recorded as negative and to the right as positive.

Sagittal radiographic parameters included: (1) pelvic incidence (PI): the angle between the line perpendicular to the sacral plate at its midpoint and the line connecting this point to the center of the femoral heads; (2) pelvic tilt (PT): the angle between the line from the center of the femoral heads to the center of the sacral superior endplate and the vertical line through the center of the femoral heads; (3) sacral slope (SS): the angle between the sacral superior endplate and the horizontal line; (4) L4-S1 lumbar lordosis: the angle between the superior endplate of L4 and the superior endplate of S1; (5) total lumbar lordosis (LL): the angle between the superior endplate of L1 and the superior endplate of S1; (6) PI-LL mismatch: the difference between PI and LL; (7) thoracic kyphosis (TK): the Cobb angle between the inferior endplate of T4 and the inferior endplate of T12; (8) T1 pelvic angle (TPA): the angle between the line from the center of T1 to the center of the femoral heads and the line from the center of the femoral heads to the center of the sacral superior endplate; and (9) sagittal vertical axis (SVA): the horizontal distance from the C7 plumb line to the posterior superior corner of S1.

**Figure 1:**
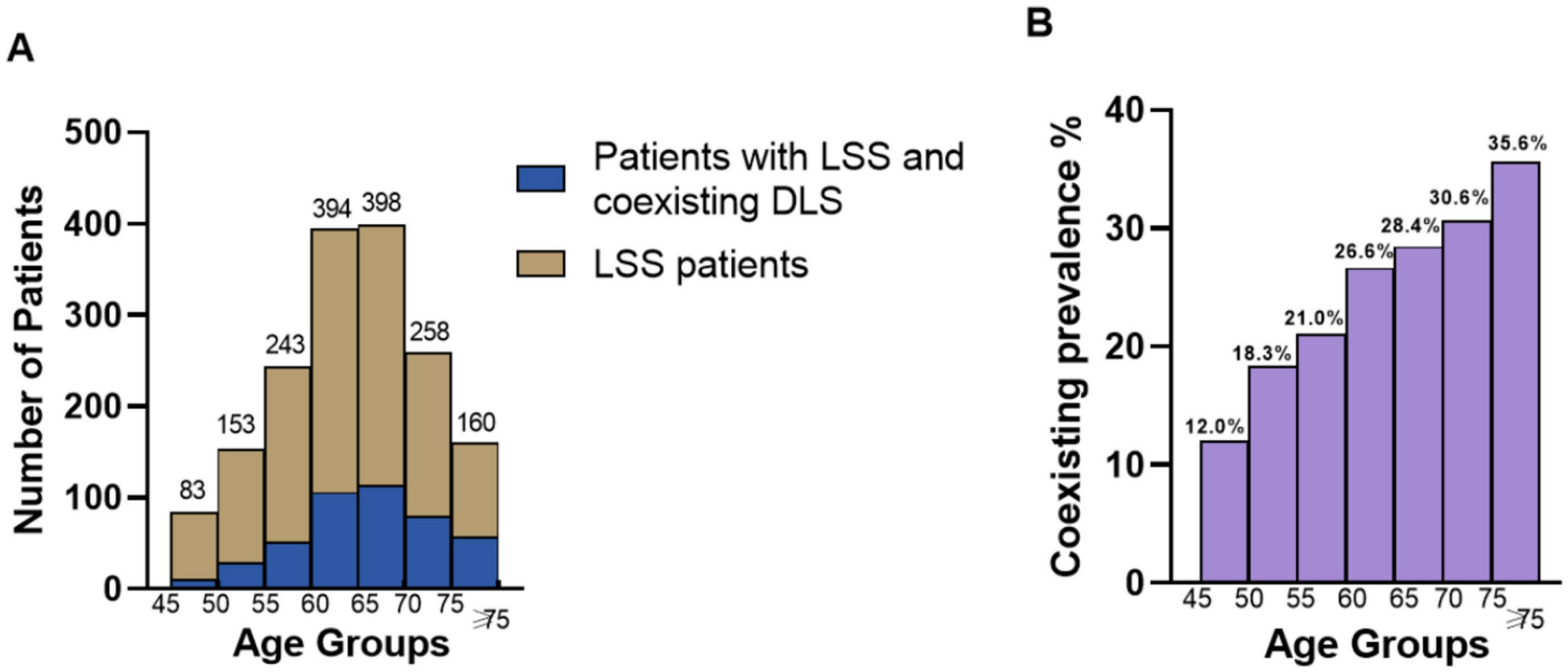
Coexisting prevalence of DLS in LSS patients in different age groups. **(A)** Number of LSS patients and LSS patients with coexisting DLS in different age groups; **(B)** Proportion of LSS patients with coexisting DLS among LSS patients in different age groups.

DLS was defined as a coronal curvature (main lumbar curve) of ≥10° measured using the Cobb method. Coronal imbalance was defined as C7PL-CSVL > 20 mm (15). Sagittal imbalance was defined as SVA > 50 mm (16). All radiological parameters were measured by two spine surgeons with more than 5 years’ clinical experience and an average value was accepted in further analysis. Intraclass correlation coefficient (ICC) between the 2 surgeons were calculated (Figure 1).

### Statistical analysis

Statistical analyses were performed using SPSS Ver. 24.0 (SPSS Inc., Chicago, IL, USA). All data were tested for normality. Spearmann correlation tests were conducted on the radiographic parameters and symptom scores. The statistical significance level was set at 0.05.

## Results

A total of 1,689 LSS patients were included in the study, with 443 having coexisting DLS, yielding a prevalence of 26.2%. Baseline clinical data was shown in Table 1.

**Table 1:** Baseline clinical data of LSS patients with coexisting DLS.

Parameter	Value
Gender (M/F)	157/286
Age	65.5 ± 7.5
BMI	26.3 ± 3.5
VAS LBP	4.8 ± 2.4
VAS leg pain	5.3 ± 2.4
ODI	56.4 ± 17.0

Data are presented as mean ± SD. VAS, visual analogue scale; LBP, low back pain; ODI, Oswestry Disability Index.

The coexisting prevalence of DLS in LSS patients increased with age, with rates of 12.0, 18.3, 21.0, 26.6, 28.4, 30.6, and 35.6% in the age groups 45–49, 50–54, 55–59, 60–64, 65–69, 70–74, and ≥75, respectively. Notable increases in coexisting were observed at ages 50, 60, and 75.

For the 443 LSS patients with coexisting DLS (157 males, 286 females; mean age 65.2 ± 7.3 years) who had an average Cobb angle of 18.0 ± 7.9°, the radiographic sagittal and coronal parameters and symptom scores are detailed in Table 2. The ICC of two observers was 0.940.

**Table 2:** Radiographic parameters of patients with LSS and coexisting DLS.

Parameter	Value
PI	51.4 ± 12.0
PT	22.3 ± 9.2
SS	29.1 ± 11.3
L4-S1	27.5 ± 11.1
LL	34.9 ± 17.2
PI-LL	18.5 ± 12.8
TK	23.9 ± 16.0
TPA	19.9 ± 9.6
SVA	45.3 ± 44.6
Cobb	18.0 ± 7.9
C7PL-CSVL	18.4 ± 15.8

Data are presented as mean ± SD. PI, pelvic incidence; PT, pelvic tilt; SS, sacral slope; LL, lumbar lordosis; TK, thoracic kyphosis; TPA, T1 pelvic angle; SVA, sagittal vertical axis; C7PL-CSVL, C7 Plumb Line-Central Sacral Vertical Line.

**Figure 2:**
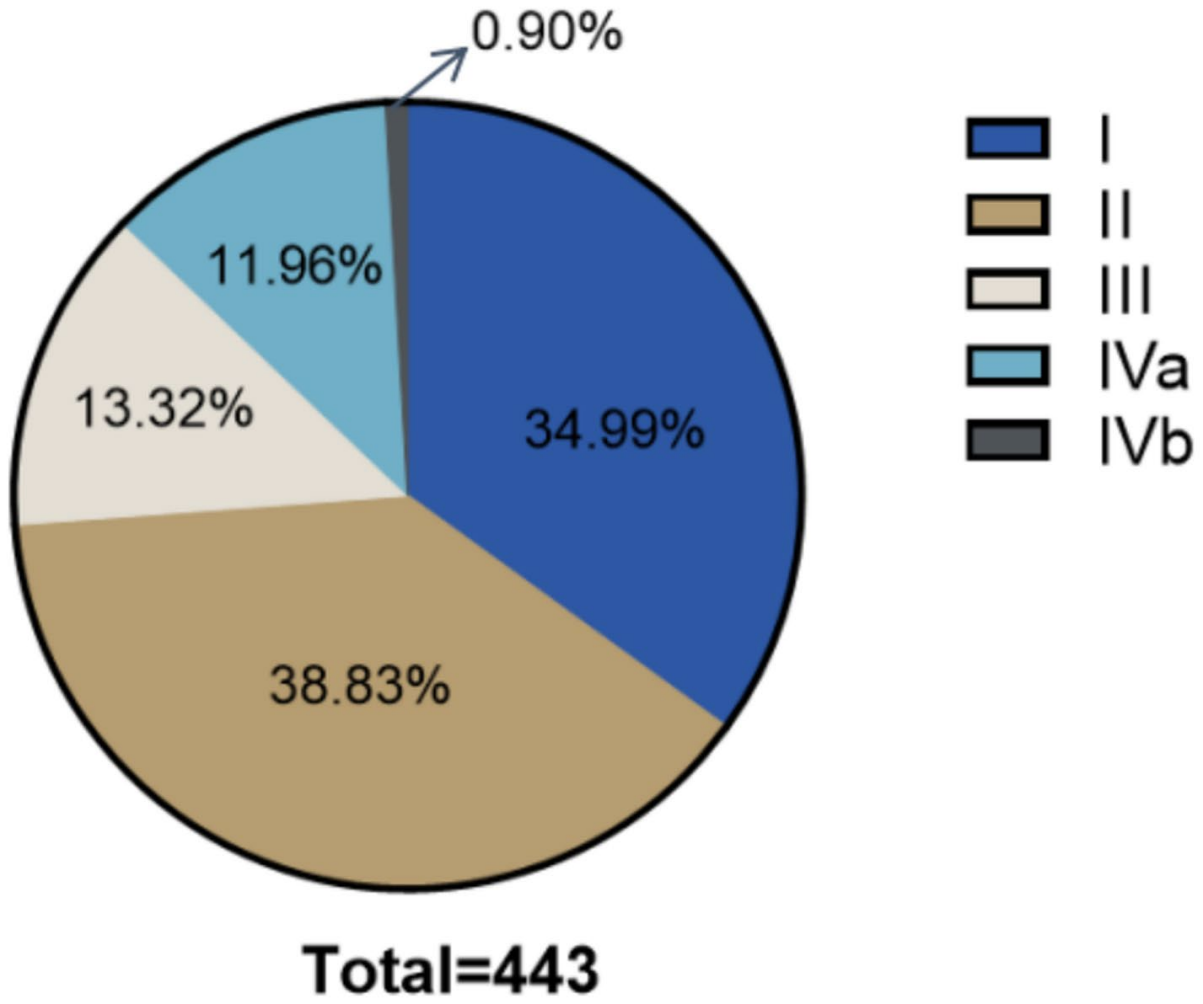
The number and proportion of cases in Berjano classification in patients with LSS and coexisting DLS.

The distribution of patients with LSS and coexisting DLS according to the Berjano classification is presented in Figure 2. Notably, Berjano type II has the highest number of cases, with 172 patients, accounting for 38.8% of the total. This is followed by type I, type III, type IVa, and type IVb, which account for 35.0, 13.3, 12.0, and 9.0%, respectively.

Among patients with LSS and coexisting DLS classified as Berjano Type II, 31 patients (18.0%) underwent fusion that extended to both sides of the coronal apex, while 141 patients (82.0%) received fusion that did not cover the apical region/fusion that did not extend to both sides of the apex. The preoperative radiographic parameters and symptom scores for these two subgroups are presented in Table 3.

**Table 3:** Comparison of preoperative radiographic parameters and symptom scores between Berjano type II patients with and without fusion extending to both sides of the coronal apex.

	Fusion extending to both sides of apex (*n* = 31)	Fusion not extending to both sides of apex (*n* = 141)	*p*
Symptomatic scores			
VAS LBP	5.8 ± 1.9	4.6 ± 2.5	0.024
VAS leg pain	5.9 ± 2.8	5.2 ± 2.5	0.175
ODI	55.9 ± 16.2	54.7 ± 16.2	0.721
Radiographic parameters			
PI	50.1 ± 9.1	52.6 ± 11.2	0.267
PT	22.2 ± 8.7	21.9 ± 8.8	0.892
SS	27.9 ± 8.3	30.6 ± 9.7	0.163
L4-S1	29.1 ± 8.3	29.2 ± 9.7	0.950
LL	34 ± 12.1	38.2 ± 12.7	0.108
PI-LL	16.1 ± 13	14.4 ± 12.9	0.524
TK	23.7 ± 15.8	25.4 ± 14.5	0.585
TPA	11 ± 10.9	9.6 ± 9.5	0.464
SVA	19.7 ± 8.8	18.9 ± 8.8	0.653
Cobb	44.5 ± 37.5	38.4 ± 38.8	0.441
C7PL-CSVL	22.2 ± 10.6	17.2 ± 7.0	0.020

Patients who underwent fusion encompassing the entire curve had significantly larger Cobb angles (22.2 ± 10.6 vs. 17.2 ± 7.0, *p* = 0.020) and higher preoperative VAS back pain scores (5.8 ± 1.9 vs. 4.6 ± 2.5, *p* = 0.024) compared to those whose fusion did not cover the whole curve. No significant differences were observed between the two groups in other radiographic parameters or symptom scores.

Table 4 shows the results of Spearman correlation tests between sagittal and coronal parameters and symptomatic scores in LSS patients with coexisting DLS. Significant correlations were found between the Cobb angle and PT (*r* = 0.171, *p* < 0.001), SS (*r* = −0.097, *p* = 0.041), LL (*r* = −0.132, *p* = 0.005), PI-LL (*r* = 0.191, *p* < 0.001), TK (*r* = −0.127, *p* = 0.008), and TPA (*r* = 0.148, *p* = 0.002). Similarly, C7PL-CSVL showed significant correlations with PT (*r* = 0.191, *p* < 0.001), LL (*r* = −0.125, *p* = 0.009), PI-LL (*r* = 0.186, *p* < 0.001), TPA (*r* = 0.198, *p* < 0.001), and SVA (*r* = 0.140, *p* = 0.003).

**Table 4:** Correlations between radiographic sagittal and coronal parameters and symptomatic scores in patients with LSS and coexisting DLS.

	Coronal parameters		Symptomatic scores		
	Cobb	C7PL-CSVL	VAS LBP	VAS leg pain	ODI
PI	0.005	0.062	0.056	0.034	0.007
PT	0.171**	0.191**	0.116*	0.07	0.130**
SS	−0.097*	−0.076	−0.012	−0.03	−0.110*
L4-S1	0.028	−0.069	0.005	−0.05	−0.114*
LL	−0.132**	−0.125**	−0.074	−0.042	−0.199**
PI-LL	0.191**	0.186**	0.141**	0.068	0.205**
TK	−0.127**	−0.092	−0.115*	−0.054	−0.122**
TPA	0.148**	0.198**	0.114*	0.073	0.187**
SVA	0.036	0.140**	0.089	0.04	0.207**
Cobb	–	–	0.158**	0.046	0.037
C7PL-CSVL	–	–	0.023	0.072	0.099*

PI, pelvic incidence; PT, pelvic tilt; SS, sacral slope; LL, lumbar lordosis; TK, thoracic kyphosis; TPA, T1 pelvic angle; SVA, sagittal vertical axis; C7PL-CSVL, C7 Plumb Line-Central Sacral Vertical Line; VAS, visual analogue scale; LBP, low back pain; ODI, Oswestry Disability Index. *p < 0.05; **p < 0.01.

Spinal radiographic parameters are more strongly associated with VAS LBP and ODI scores, whereas no significant correlations were observed with VAS leg pain. VAS LBP was significantly correlated with the Cobb angle (*r* = 0.158, *p* = 0.001), PT (*r* = 0.116, *p* = 0.014), PI-LL (*r* = 0.141, p = 0.003), TK (*r* = −0.115, *p* = 0.015), and TPA (*r* = 0.102, *p* = 0.016). ODI showed significant correlations with C7PL-CSVL (*r* = 0.099, *p* = 0.038), PT (*r* = 0.130, *p* = 0.006), SS (*r* = −0.110, *p* = 0.020), L4-S1 (*r* = −0.114, *p* = 0.017), LL (*r* = −0.199, *p* < 0.001), PI-LL (*r* = 0.205, *p* < 0.001), TK (*r* = −0.122, *p* = 0.010), TPA (*r* = 0.187, *p* < 0.001), and SVA (*r* = 0.207, *p* < 0.001).

The relationship between coronal and sagittal imbalance in LSS patients with coexisting DLS is depicted more intuitively in Table 5. The overall sagittal imbalance rate (SVA > 50 mm) was 41.1%. Notably, as coronal imbalance increased, sagittal imbalance also worsened: for C7PL-CSVL ≤ 20 mm, the rate was 37.3%; for C7PL-CSVL between 20 mm and 40 mm, it rose to 47.2%; and for C7PL-CSVL ≥ 40 mm, it reached 51.1%.

**Table 5:** The sagittal imbalance rate in patients with LSS and coexisting DLS with different degrees of coronal imbalance.

	Patients with SVA > 50 mm	Number of patients	Sagittal imbalance rate
C7PL-CSVL ≤ 20 mm	109	292	37.3%
20 mm < C7PL-CSVL ≤ 40 mm	50	106	47.2%
C7PL-CSVL ≥ 40 mm	23	45	51.1%
Total	182	443	41.1%

SVA, sagittal vertical axis; C7PL-CSVL, C7 Plumb Line-Central Sacral Vertical Line.

## Discussion

Through a relatively large patient cohort, this study found that the co-prevalence of DLS in patients with LSS requiring surgery was 26.2%. In clinical practice, the choice of surgical strategy for the specific patient population could be more complex and controversial (8, 9). Therefore, it is essential for spine surgeons to fully recognize the unique clinical characteristics of these patients. While the relationship between spinopelvic parameters and clinical symptoms has been well established in adult spinal deformity populations, its application to surgical LSS patients with coexisting DLS—a population defined by predominant neurogenic symptoms with secondary deformity—remains underexplored.

In previous studies, the prevalence of DLS in patients with LSS ranged from 11.6 to 21.9%, showing significant variability (10–14). The prevalence of DLS in our study was higher than previously reported. Further, with advancing age, the prevalence of DLS increased significantly, suggesting a strong association between aging and the development of DLS in LSS patients.

Our study describes the preoperative spinopelvic profile of LSS patients with coexisting DLS, observing a significant tendency for sagittal imbalance—manifested by increased PT, SVA, and PI-LL mismatch—in addition to their coronal scoliosis. These radiographic parameters significantly correlated with patient-reported pain and neurological function. Specifically, VAS LBP was significantly correlated with the Cobb angle, PT, PI-LL, TK and TPA, while ODI was significantly associated with C7PL-CSVL, PT, SS, L4-S1, LL, PI-LL, TK, TPA and SVA. The results aligned with previous findings by Schwab et al. (17–19) in adult spinal deformity patients and suggested that the structural deformity could be a key contributor to the more severe symptoms and neurological dysfunction seen in this LSS subgroup. These findings suggest that for elderly patients undergoing surgery for LSS, focusing solely on the neural compression at the stenotic segment may be insufficient, and a preoperative evaluation of the overall spinal alignment is essential.

In contrast, leg pain showed no significant correlation with any X-ray parameters. This finding is consistent with previous studies reporting that Cobb angle correlates with back pain but not with leg pain in spinal deformity populations (20). The severity of coronal curvature does not directly determine the degree of focal neural compression, which is the primary cause of radicular leg pain. Meanwhile, coronal deformity in DLS is often accompanied by sagittal imbalance, which is an important contributor to axial back pain. Moreover, our data revealed a clear trend where the severity of coronal imbalance (C7PL-CSVL) was associated with a higher incidence of sagittal imbalance (SVA > 50 mm), which rose from 37.3% in the mildest group to 51.1% in the most severe group. This finding highlights the three-dimensional nature of the deformity. Therefore, a comprehensive evaluation of spinal alignment in clinical practice should include a simultaneous assessment of balance in multiple planes.

Patients with LSS and coexisting DLS occupy a clinical gray area between degeneration and deformity, making surgical strategy particularly controversial. Although previous studies reported that decompression alone may be with acceptable outcomes for LSS patients with coexisting DLS (12), for patients with significant imbalance, restoring a suitable spinopelvic alignment would still be necessary to prevent the progression of scoliosis and achieve better clinical outcome (13, 21). However, given the increased medical expenses and surgical risks inherent to corrective surgery, determining how to restore spinal balance with minimal cost presents another challenge in clinical practice.

To address the issue, Berjano et al. (10) proposed a classification system used to categorize different types of DSD and to guide personalized surgical strategies. However, few studies have verified the effectiveness of the Berjano classification as a preoperative tool for guiding clinical treatment in patients with both LSS and DLS. Therefore, the present study explored the distribution of Berjano subtypes in this specific patient group. Our results suggest that Type II and Type I are relatively more common, accounting for 35.0 and 38.8%, respectively, while Type III, Type IVa, and Type IVb are less common, accounting for 13.3, 12.0, and 9.0%, respectively. The high proportion of Type II patients in our cohort suggests that this subgroup deserves particular attention in future studies on optimal surgical strategies.

Our subgroup analysis of Berjano Type II patients further supports this concern. Only 18.0% of Type II patients underwent fusion encompassing the entire coronal curve, and these patients had significantly larger Cobb angles and more severe back pain than those receiving less extensive fusion. This indicates that in clinical practice, surgeons reserve the recommended long fusion for Type II patients with greater deformity severity and symptoms, while treating milder cases with more limited procedures. For future research, it is crucial to accurately assess the respective impacts of local neural compression versus global spinal imbalance in patients with LSS and coexisting DLS. This is essential for developing accurate, reliable, and individualized management and surgical strategies, particularly for patients with Berjano Type II deformity.

The limitations of this study include (1) its retrospective design; (2) the single-center design and single-ethnicity cohort; (3) although MRI was used to confirm the diagnosis of LSS, the absence of quantitative MRI-based stenosis grading (e.g., Schizas classification) limits our ability to directly correlate neural compression severity with leg pain symptoms; this will be addressed in future studies; and (4) the need for longer follow-up periods in prospective studies to confirm our findings. The strengths of this study are (1) a large sample size; (2) comprehensive radiographic parameters and symptom scores; and (3) whether interventions for LSS with coexisting DLS should be applied, the details of these interventions, as well as the long-term prognosis of the patients, remain to be explored in future follow-up studies.

## Data Availability

The datasets presented in this article are not readily available because the data contain sensitive patient information and are protected by institutional ethics policies and national data privacy laws. Requests to access the datasets should be directed to the corresponding author and will require approval from the originating institutional ethics committee and the execution of a data transfer or use agreement. Requests to access the datasets should be directed to puh3liweishi@163.com.
